# Clinical utility of brachial-ankle pulse wave velocity in the prediction of cardiovascular events in diabetic patients

**DOI:** 10.1186/s12933-014-0128-5

**Published:** 2014-09-05

**Authors:** Naoto Katakami, Takeshi Osonoi, Mitsuyoshi Takahara, Miyoko Saitou, Taka-aki Matsuoka, Yoshimitsu Yamasaki, Iichiro Shimomura

**Affiliations:** Department of Metabolic Medicine, Osaka University Graduate School of Medicine, 2-2, Yamadaoka, Suita, 565-0871 Osaka Japan; Department of Metabolism and Atherosclerosis, Osaka University Graduate School of Medicine, Osaka, Japan; Naka Memorial Clinic, Naka City, Japan

**Keywords:** Brachial-ankle pulse wave velocity (baPWV), Carotid intima-media thickness (carotid IMT), Cardiovascular risk, Diabetes mellitus

## Abstract

**Background:**

Brachial-ankle pulse wave velocity (baPWV) is a method to estimate arterial stiffness, which reflects the stiffness of both the aorta and peripheral artery; it would be applicable to general practice, since its measurementis automated. The aim of this study was to evaluate whether baPWV can be predictors of future cardiovascular events (CVE) in diabetic patients.

**Methods:**

We prospectively evaluated the association between baPWV or carotid intima-media thickness (carotid IMT) at baseline and new onset of CVE in 1040 type 2 diabetic patients without CVE. The predictability of baPWV and/or carotid IMT for identifying patients at high risk for CVE was evaluated by time-dependent receiver-operating-characteristic (ROC) curve analysis.

**Results:**

During a median follow-up of 7.5 years, 113 had new CVD events. The cumulative incidence rates of CVE were significantly higher in patients with high baPWV values (≥1550 cm/s) as compared to those with low baPWV values (<1550 cm/s) (*p* < 0.001, log-rank test). Similarly, the cumulative incidence rate of CVE was significantly higher in patients with higher maximum carotid IMT (maxIMT) values (≥1.0 mm) as compared to those with lower maxIMT values (<1.0 mm) (*p* < 0.001, log-rank test). Subjects with both “high PWV” and “high IMT” had a significantly higher risk of developing CVE as compared to those with either “high PWV” or “high IMT,” as well as those with neither. A multivariate Cox proportional hazards regression model revealed that both baPWV (HR = 1.30, [95%CI: 1.07-1.57]; *p* = 0.009) and maxIMT (HR = 1.20, [95%CI: 1.01-1.41]; *p* = 0.033) were independent predictors for CVE, even after adjustment for the conventional risk factors. Time-dependent ROC curve analyses revealed that the addition of maxIMT to the Framingham risk score resulted in significant increase in AUC (from 0.60 [95%CI: 0.54-0.67] to 0.63 [95%CI: 0.60-0.82]; *p* = 0.01). Notably, the addition of baPWV to the Framingham risk score and maxIMT resulted in further and significant (*p* = 0.02) increase in AUC (0.72 [95%CI: 0.67-0.78]).

**Conclusions:**

Evaluation of baPWV, in addition to carotid IMT and conventional risk factors, improved the ability to identify the diabetic individuals with high risk for CVE.

**Electronic supplementary material:**

The online version of this article (doi:10.1186/s12933-014-0128-5) contains supplementary material, which is available to authorized users.

## Background

Because cardiovascular disease is the main cause of death and impairment of quality of life in diabetic patients, early identification of individuals at high risk for cardiovascular events (CVE) and subsequent rapid interventions are important. Therefore, cardiovascular risk assessment based on conventional risk factors, such as obesity, hypertension, and dyslipidemia, is recommended for predicting CVE. However, validation studies showed that this approach had only moderate performance [1-3].

Recently, many studies have indicated that arterial stiffness, which depends on the structural and geometric properties of the arterial wall, plays a critical role in the pathogenesis of atherosclerosis and CVE [4-6]. Among several indices for quantifying the level of arterial stiffness, carotid-femoral pulse wave velocity (cfPWV) is a test in common use. Several studies have demonstrated that higher cfPWV levels are associated with increased risk of CVE and premature death [4,5,7-9], and it has therefore been considered as the gold standard for evaluation of central aortic stiffness. However, accurate measurement of cfPWV levels requires specialized training and a cumbersome procedure, and it has not been applied in routine clinical practice.

Brachial-ankle pulse wave velocity (baPWV) is another method to estimate arterial stiffness. It reflects the stiffness of both the aorta and peripheral arteries in an arm and a leg, and would be more applicable to general practice since its measurement, which uses a separate cuff for each limb, is automated and easier to perform than cfPWV [10-12]. Because baPWV was shown to be correlated with cfPWV in several cross-sectional studies [13,14], it could also be a good predictor of CVE. Indeed, several studies have shown that baPWV is an independent predictor of the presence of coronary artery disease [15], myocardial injury [16], and future CVE [17,18]. However, it remains unclear whether measurement of baPWV can add prognostic information beyond conventional cardiovascular risk markers in patients with type 2 diabetes.

On the other hand, many studies have revealed close associations between carotid atherosclerosis and cardiovascular disease. Carotid ultrasonography is a simple and non-invasive procedure that has allowed clinicians to visualize the characteristics of the carotid wall and lumen surfaces to quantify the severity of atherosclerosis. In particular, carotid intima-media thickness (IMT) is a useful marker of the progression of atherosclerosis throughout the body, and has been shown to be a predictor of CVE [17,19-25]. Thus, carotid IMT is considered one of the most appropriate screening tools for identifying high-risk individuals.

On this background, the present study prospectively evaluated whether measurement of baPWV can add prognostic information beyond conventional cardiovascular risk markers in patients with asymptomatic type 2 diabetes. Furthermore, this study also evaluated whether the addition of baPWV values to conventional risk factors and maximum carotid IMT (maxIMT) can improve the predictability of CVE in this population.

## Methods

### Subjects

The present study was based on data obtained from the Study of Order-Made multiple Risk Factor Intervention Trial (OMRFIT), which is an ongoing multicenter cohort study to investigate the genetic risk factors for diabetic complications, and has recruited more than 4000 diabetic patients. The present study included a total of 1040 type 2 diabetic patients who were registered at either Osaka University Hospital or Naka Memorial Clinic, where the measurement of baPWV and ankle brachial index (ABI) was performed routinely. Patients were enrolled during January-December 2005 and fulfilled the following criteria at baseline: (I) diagnosis of type 2 diabetes based on criteria of the Japan Diabetes Society (early-morning fasting plasma glucose (PG) ≥126 mg/dL, two-hour PG after 75 g glucose load ≥200 mg/dL, or casual plasma glucose ≥200 mg/dL) [26], (II) exclusion of a diagnosis of coronary heart disease (CHD), cerebral infarction, or peripheral artery disease (PAD) based on medical interviews, physical examinations, and screening examinations, including electrocardiogram, chest X-ray, and ABI, (III) measurement of baPWV. The study protocol was approved by the committees on the ethics of human research of Osaka University Graduate School of Medicine. Written informed consent was obtained from all the participants after they received a full explanation of the study.

### Clinical and biochemical analyses

Clinical and biochemical data were collected at baseline. A structured questionnaire was used to determine medical history including duration of diabetes, current medication use, and smoking status. Smoking status was evaluated as a value of 0 or 1 when Brinkman’s Index (the number of cigarettes per day x smoking years) was less than 200 or higher than 200, respectively. Blood pressure was measured at rest with a mercury and/or automated sphygmomanometer. Measurement of ABI was performed with subjects in the supine position after at least 5 min of rest, using a volume-plethysmographic apparatus (BP-203RPE II form PWV/ABI; Omron Healthcare Co., Ltd., Kyoto, Japan). Fasting blood samples were collected and hemoglobin A1c (HbA1c), serum total, LDL, and HDL cholesterol, and serum triglyceride (TG) levels were measured using standard laboratory protocols. The determination of hypertension (defined as systolic blood pressure (SBP) ≥130 mmHg or diastolic blood pressure (DBP) ≥80 mmHg or anti-hypertensive medication use) and dyslipidemia (defined as serum LDL cholesterol ≥3.1 mmol/L (120 mg/dl) or serum TG ≥1.7 mmol/L (150 mg/dl) or HDL-cholesterol <1.0 mmol/L (40 mg/dl) or lipid-lowering medication use) was based on the criteria of the Japan Diabetes Society [26]. The risk of CVD was also estimated, using the Framingham D’Agostino equations [26], with gender, age, total cholesterol, HDL cholesterol, systolic blood pressure, antihypertensive medication use, current smoking status, and diabetes status included as risk factors in the model.

### Measurement of baPWV

Measurement of baPWV was performed using the same volume-plethysmographic apparatus as for ABI (BP-203RPE II form PWV/ABI), with subjects in the supine position after at least 5 min of rest [10,11,13]. Four oscillometric cuffs, each connected to a plethysmographic sensor that determined volume pulse form and to an oscillometric pressure sensor that measured blood pressure, were wrapped on both the brachia and ankle and two electrocardiogram electrodes were placed on each wrist They were simultaneously pressurized to the approximate value of the patient’s diastolic pressure so that the pulse volume waveforms could be recorded using semiconductor pressure sensors. The distance between sampling points of baPWV was calculated automatically according to the height of the subject. The path length from the suprasternal notch to the ankle (La) was calculated as: La = 0.8129*height (in cm) + 12.328. The path length from the suprasternal notch to the brachium (Lb) was calculated as: Lb = 0.2195*height - 2.0734. The baPWV was calculated according to the following formula: baPWV = (La - Lb)/Tba, where Tba was the time interval between the wave front of the brachial waveform and that of the ankle waveform [13]. Two simultaneous measurements of baPWV were recorded, on the right side and left side, respectively, and the higher of these readings was used as the representative value for each individual.

### Measurement of Carotid IMT

B-mode ultrasonography of the carotid artery was performed using an ultrasound machine (SDU 2200, Shimazdu Medical Inc., Japan) with a 7.5-MHz linear transducer. In accordance with the guidelines of the Japan Society of Ultrasonics [27], all scanning was conducted by experienced laboratory physicians using the same ultrasound system and the same measuring method. Scanning of the extracranial common carotid artery, the carotid bulb, and the internal carotid artery in the neck was performed bilaterally from three different longitudinal projections (i.e., anterior-oblique, lateral, and posterior-oblique). The carotid IMT was measured as the distance from the leading edge of the first echogenic line to the leading edge of the second echogenic line using computer software (Intima-scope; Media Cross Inc., Tokyo, Japan). The thickest points of IMT (including plaque lesions) in the common carotid artery, the carotid bulb, and the internal carotid artery were measured separately, and the highest value among them was defined as maxIMT and used as the representative value for each individual [27].

### Assessment of cardiovascular events

The prespecified outcome during the follow-up period was the first occurrence of CVE, which included any coronary heart disease (CHD) event (coronary death, myocardial infarction, angina, and coronary revascularization), any cerebrovascular event (ischemic stroke, hemorrhagic stroke, and transient ischemic attack), peripheral artery disease (PAD), and heart failure. The occurrence of acute coronary syndrome, stroke, heart failure, sudden death and cardiac death were defined as the major CVE. Diagnosis of occurrence of a CHD event was performed by cardiologists based on the clinical symptoms, characteristic ECG changes, cardiac enzyme levels, and findings in coronary angiography and/or echocardiography, according to established guidelines [28,29]. An ischemic stroke event was defined as a validated definite or probable hospitalized atherothrombotic, cardioembolic, lacunar, or other type of ischemic stroke, diagnosed by neurosurgical experts based on clinical symptoms and neuroimaging findings, according to the National Institute of Neurological Disorders and Stroke (NINDS) III classification [30]. The presence of lower extremity PAD was defined as the highest stenosis observed on either the right or left side. An ABI <0.9 was diagnostic of occlusive arterial disease in patients with or without symptoms. The measurement of ABI was performed approximately annually during the follow-up period and subjects whose ABI value decreased to below 0.9 were diagnosed as developing PAD. Diagnosis of chronic heart failure was performed by cardiologists based on the clinical symptoms, characteristic chest X-ray changes, plasma BNP levels, and findings in other tests, including echocardiography and cardiac catheter test, according to the Guidelines for Treatment of Chronic Heart Failure published by the Japanese Circulation Society [31,32].

All patients were followed up at each hospital visit, or by telephone if necessary, and the occurrence of medical events was determined. For potential new CVE, additional information, including the results of imaging and other diagnostic procedures, was obtained for confirmation, and all causes of death were confirmed by hospital records. For participants with incident CVE, follow-up duration was defined as the period between the baseline clinic visit and the date of the first CVE. For participants with no CVE, follow-up continued until the date of death or December 31, 2012, or until the date of last contact. Patients were allowed to use any concurrent treatment.

### Statistical analyses

All values are reported as mean ± SD, or median for continuous variables, or number with percentage in parentheses for categorical variables. The occurrence of CVE and major CVE were plotted using the Kaplan-Meier method and differences between the groups were assessed by a log-rank test. Univariate and multivariate Cox proportional hazards regression models were used to determine the association of each variable with the outcome, and hazard ratios (HRs) and 95% confidence interval (CI) were reported. Statistically significant variables in the unadjusted analyses were entered into the multivariate model to reveal the independent impact on the outcome.

The ability of variables to predict CVE and major CVE were examined by time-dependent receiver-operating-characteristic (ROC) curve analysis [33]. Curves were generated from models of the prediction of risk using Framingham risk score (FRS) alone, or with carotid IMT, or with carotid IMT and baPWV. For all tests, *p* < 0.05 was considered statistically significant. The statistical analyses were performed using SPSS version 19.0 J (SPSS, Chicago, IL).

## Results

### Patient characteristics

The baseline characteristics of the overall study population and of subgroups identified according to median baPWV level (median: 1550 cm/s) and median value of maxIMT (1.0 mm) are shown in Table 1. Among a total of 1040 patients (males, 65.0%; age, 58.9 ± 9.6 years; diabetes duration, 4.8 ± 4.4 years; HbA1c, 7.2 ± 1.1%), 767 (73.8%) patients had hypertension and 728 (70.0%) had dyslipidemia. Mean age, HbA1c levels, duration of diabetes, systolic blood pressure, and FRS were significantly higher in patients with higher baPWV values (≥1550 cm/s, n = 520) compared to those with lower baPWV values (<1550 cm/s, n = 520) (p < 0.05). Frequencies of hypertension and antidiabetic and antihypertensive medication use were also significantly higher in subjects with higher baPWV (p < 0.05). There was no significant difference between the two groups regarding gender, smoking habit, BMI, diastolic blood pressure, serum total, HDL and LDL cholesterol concentrations, triglyceride, and creatinine levels. These findings were consistent with previous reports, suggesting that high baPWV itself is closely associated with cardiovascular risk factors.

**Table 1:** **Baseline characteristics of the overall study population and classified according to the medians of baPWV and maxIMT**

**Parameters**	**All patients (n = 1040)**	**baPWV**		**maxIMT**	
		**<1550 cm/s (n = 520)**	**≥1550 cm/s (n = 520)**	**<1.0 mm (n = 460)**	**≥1.0 mm (n = 580)**
Gender (male,%)	65.0	65.4	64.6	62.6	66.9
Age (years)	58.9 ± 9.6	55.2 ± 10.1	62.5 ± 7.6*	55.1 ± 10.0	61.8 ± 8.2*
Smoking habit	47.9	47.5	48.3	45.0	50.2
Body mass index (kg/m^2^)	23.9 ± 3.5	24.0 ± 3.9	23.8 ± 3.1	23.8 ± 3.9	24.0 ± 3.2
HbA1c (%)	7.2 ± 1.1	6.7 ± 1.1	6.8 ± 1.1‡	6.8 ± 1.1	6.8 ± 1.2
Duration of diabetes (years)	4.8 ± 4.4	4.2 ± 3.2	5.5 ± 5.2*	4.4 ± 3.2	5.1 ± 5.1‡
Systolic BP (mmHg)	131 ± 17	126 ± 16	137 ± 17*	128 ± 16	133 ± 17*
Diastolic BP (mmHg)	80 ± 11	80 ± 11	81 ± 11	81 ± 11	80 ± 12
Presence of hypertension (%)	73.8	63.1	84.6*	69.9	77.1†
Total cholesterol (mg/dl)	188 ± 29	188 ± 29	187 ± 29	188 ± 29	187 ± 29
HDL cholesterol (mg/dl)	59 ± 16	59 ± 16	58 ± 17	61 ± 18	57 ± 15*
LDL cholesterol (mg/dl)	106 ± 24	106 ± 23	105 ± 25	104 ± 23	107 ± 24
Triglyceride (mg/dl)	120 ± 89	120 ± 102	121 ± 73	120 ± 100	121 ± 78
Presence of dyslipidemia (%)	70.0	69.4	70.6	69.6	70.3
Serum creatinine (mg/dl)	0.77 ± 0.36	0.75 ± 0.28	0.79 ± 0.43	0.74 ± 0.22	0.79 ± 0.45‡
Framingham risk score (%)	27.4 ± 16.8	21.3 ± 14.3	33.4 ± 17.0*	21.9 ± 14.9	31.7 ± 17.0*
Administration of					
Anti-diabetic drugs (%)	70.3	66.3	74.2†	68.7	71.6
Anti-hypertensive drugs (%)	38.8	27.7	49.8*	32.4	43.8*
Anti-hyperlipidemic drugs (%)	40.6	39.6	41.5	40.4	40.7

Data are shown as% or means ± SD.

*p < 0.001 for bivariate comparisons between patients classified according to the medians of the baPWV levels and the maxIMT.

†p < 0.01 for bivariate comparisons between patients classified according to the medians of the baPWV levels and the maxIMT.

‡p < 0.05 for bivariate comparisons between patients classified according to the medians of the baPWV levels and the maxIMT.

Mean age, duration of diabetes, systolic blood pressure, serum creatinine levels, and FRS were significantly higher and serum HDL cholesterol levels were significantly lower in patients with higher maxIMT values (≥1.0 mm, n = 580) compared to those with lower maxIMT values (<1.0 mm, n = 460) (p < 0.05). Frequencies of hypertension and antihypertensive medication were also significantly higher in subjects with higher maxIMT (p < 0.05). There was no significant difference between the two groups regarding the other clinical parameters.

During a median follow-up of 7.5 years, 113 of the 1040 patients (10.9%) had new CVE (40 CHD events, 56 cerebrovascular events, 14 PAD, and 3 heart failure cases). Among them, 66 cases (6 acute coronary syndrome, 56 stroke, 2 heart failure cases and 2 cardiac sudden deaths) were defined as the major CVE. During the observation period, 20 participants died (2 were cardiac sudden deaths, 8 were cancer deaths, and the others were various causes of deaths).

Univariate Cox regression analysis showed that age (HR per 1 year = 1.08 [95%CI 1.05-1.10]; p < 0.001), duration of diabetes (HR per 1 year = 1.05 [95%CI 1.02-1.09]; p = 0.001), presence of hypertension at baseline (HR = 1.61 [95%CI 1.00-2.58]; p = 0.049), baseline systolic blood pressure (HR per 1 mmHg = 1.02 [95%CI 1.01-1.03]; p = 0.004), serum creatinine concentration (HR per 1 mg/dl = 1.54 [95%CI 1.17-2.03]; p = 0.002), and FRS (HR per 1 SD = 1.40 [95%CI 1.18-1.65]; p < 0.001) were significantly associated with the development of CVE. Other clinical and laboratory variables, including gender, smoking habit, BMI, HbA1c, diastolic blood pressure, serum LDL cholesterol, HDL cholesterol, and triglyceride concentration, presence of dyslipidemia, and administration of anti-diabetic, anti-hypertensive, and anti-hyperlipidemic drugs were not associated with the development of CVE (Additional file 1: Table S1).

### Association between baPWV and cardiovascular events

**Figure 1:**
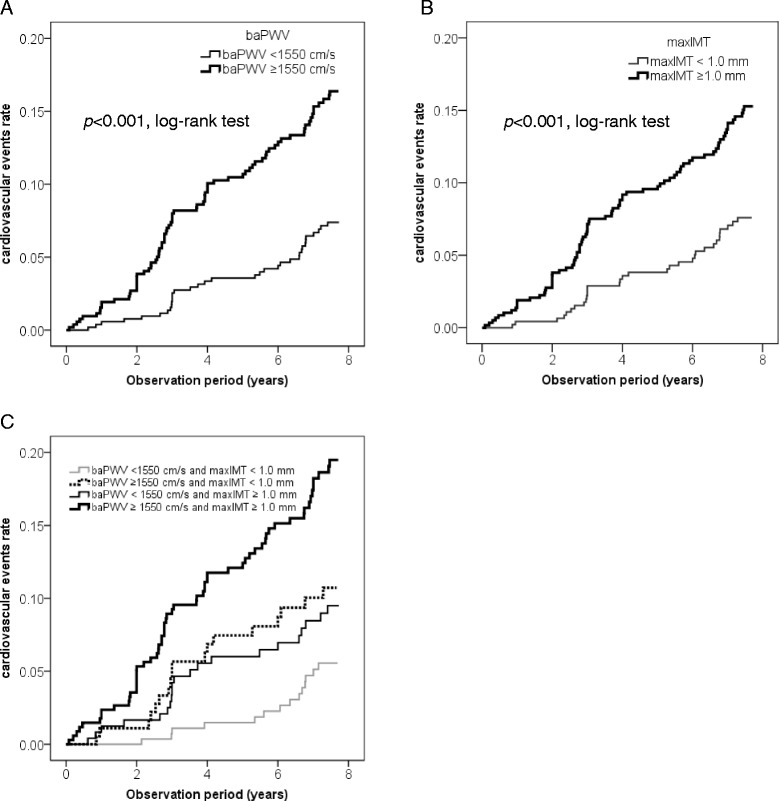
**Kaplan-Meier curves depicting the cumulative probability of cardiovascular events. A** The risk for cardiovascular events was significantly greater in patients with higher baPWV values (bold line) (≥1550 cm/s, n = 520) compared to those with lower baPWV values (thin line) (<1550 cm/s, n = 520) (*p* < 0.001, log-rank test). **B** The risk for cardiovascular events was significantly greater in patients with higher maxIMT values (bold line) (≥1.0 mm, n = 580) compared to those with lower maxIMT values (thin line) (<1.0 mm, n = 460) (*p* < 0.001, log-rank test). **C** The cumulative incidence rate of cardiovascular events was significantly greater in the patients with “high baPWV and low maxIMT (baPWV ≥1550 cm/s and maxIMT <1.0 mm, n = 181)” (dotted line) compared to those with “low baPWV and low maxIMT (baPWV <1550 cm/s and maxIMT <1.0 mm, n = 279)” (thin gray line) (p = 0.030, log-rank test). The patients with “low baPWV and high maxIMT (baPWV <1550 cm/s and maxIMT ≥1.0 mm, n = 241)” (thin black line) also showed a tendency towards a higher risk compared to those with “low baPWV and low maxIMT” (p = 0.071, log-rank test). The cumulative incidence rate of cardiovascular events was significantly higher in the patients with “high baPWV and high maxIMT (baPWV ≥1550 cm/s and maxIMT ≥1.0 mm, n=580)” (bold black line) compared to the other 3 groups.

As shown in Figure 1A, the cumulative incidence rate of CVE was significantly higher in patients with higher baPWV values compared to those with lower baPWV values (*p* < 0.001, log-rank test). Table 2 shows the results of Cox proportional hazards regression for baPWV analyzed as a continuous variable as a predictor of CVE. A univariate Cox proportional hazards regression model revealed that the baPWV was a significant predictor of CVE, with a 67% excess risk for each 1 SD increment (HR per 1 SD increment = 1.67 [95%CI: 1.44-1.93]; p < 0.001). A multivariate Cox proportional hazards regression model, in which age and gender were included as independent variables, also revealed that the baPWV was an independent predictor of CVE (HR = 1.46 [95%CI: 1.23-1.73]; p < 0.001), even after adjustment for gender and age. Next, to confirm that the baPWV was an independent predictor of CVE even after adjustment for the other statistically significant variables in the unadjusted analyses, another multivariate Cox proportional hazards regression model, which included factors for gender, age, duration of diabetes, presence of hypertension at baseline, baseline systolic blood pressure, serum creatinine level, and FRS, was performed. This analysis revealed that baPWV was remained independently associated with CVE after adjustment for these factors (HR = 1.32 [95%CI: 1.09-1.59]; p = 0.005). Furthermore, another multivariate Cox proportional hazards regression model, adjusted for conventional risk factors and baseline therapies (gender, age, smoking habit, BMI, HbA1c, duration of diabetes, systolic blood pressure, HDL cholesterol, LDL cholesterol, triglyceride, serum creatinine, administration of anti-diabetic drugs, anti-hypertensive drugs, and anti-hyperlipidemic drugs) also showed that baPWV was independently associated with CVE (HR = 1.39 [95%CI: 1.10-1.61]; p = 0.004).

**Table 2:** **Relative risk of cardiovascular events**

	**HR (95% CI)**	***p*** **value**
baPWV (per 1 SD)		
Model 1	1.67 (1.44-1.93)	<0.001
Model 2	1.46 (1.23-1.73)	<0.001
Model 3	1.32 (1.09-1.59)	0.005
Model 4	1.35 (1.11-1.64)	0.003
Model 4 + maxIMT	1.33 (1.09-1.62)	0.004
maxIMT (per 1 SD)		
Model 1	1.46 (1.28-1.66)	<0.001
Model 2	1.29 (1.11-1.50)	0.001
Model 3	1.22 (1.03-1.44)	0.019
Model 4	1.22 (1.03-1.46)	0.022
Model 4 + baPWV	1.21 (1.01-1.44)	0.036

Cox proportional hazards regression analyses unadjusted (Model 1) and adjusted for the following covariates:

Model 2, adjusted for gender and age.

Model 3, adjusted for gender and the other statistically significant variables in the unadjusted analyses (age, duration of diabetes, presence of hypertension at baseline, baseline systolic blood pressure, serum creatinine level, and Framingham risk score).

Model 4, adjusted for the conventional risk factors and baseline therapies (gender, age, smoking habit, BMI, HbA1c, duration of diabetes, systolic blood pressure, HDL cholesterol, LDL cholesterol, triglyceride, serum creatinine level, administration of anti-diabetic drugs, anti-hypertensive drugs, and anti-hyperlipidemic drugs).

Analysis based on new onset of the “major CVE” showed that the cumulative incidence rate was also significantly higher in patients with higher baPWV values compared to those with lower baPWV values (p = 0.001, log-rank test) (Additional file 2: Figure S1A.) Furthermore, a multivariate Cox proportional hazards regression model adjusted for conventional risk factors and baseline therapies (gender, age, smoking habit, BMI, HbA1c, duration of diabetes, systolic blood pressure, HDL cholesterol, LDL cholesterol, triglyceride, serum creatinine level, administration of anti-diabeteic drugs, anti-hypertensive drugs, and anti-hyperlipidemic drugs) showed that baPWV remained independently associated with the major CVE (HR = 1.32 [95%CI: 1.08-1.78]; p = 0.011) (Additional file 1: Table S2).

### Association between carotid IMT and cardiovascular events

As shown in Figure 1B, the cumulative incidence rate of CVE was significantly higher in patients with higher maxIMT values (≥1.0 mm) compared to those with lower maxIMT values (<1.0 mm) (*p* < 0.001, log-rank test). A univariate Cox proportional hazards regression model revealed that the maxIMT was a significant predictor of CVE, with a 46% excess risk for each 1 SD increment (HR per 1 SD increment = 1.46 [95%CI: 1.28-1.66]; p < 0.001). Multivariate Cox proportional hazards regression model adjusted for conventional risk factors and/or baseline therapies (gender, age, smoking habit, BMI, HbA1c, duration of diabetes, systolic blood pressure, HDL cholesterol, LDL cholesterol, triglyceride, serum creatinine, administration of anti-diabetic drugs, anti-hypertensive drugs, and anti-hyperlipidemic drugs) also revealed that maxIMT was independently associated with CVE (Table 2).

The cumulative incidence rate of the major CVE was also significantly higher in patients with higher maxIMT values compared to those with lower maxIMT values (*p* = 0.003, log-rank test) (Additional file 3: Figure S1B). A multivariate Cox proportional hazards regression model revealed that the maxIMT was a significant predictor of the major CVE (HR per 1 SD increment = 1.30 [95%CI: 1.10-1.55]; p = 0.002), even after adjustment for the FRS. However, another model adjusted for the conventional risk factors and baseline therapies (gender, age, smoking habit, BMI, HbA1c, duration of diabetes, systolic blood pressure, HDL cholesterol, LDL cholesterol, triglyceride, serum creatinine, administration of anti-diabetic drugs, anti-hypertensive drugs, and anti-hyperlipidemic drugs) revealed that maxIMT was no longer a significant predictor of the major CVE (Additional file 1: Table S2).

Next, we classified study patients into 4 groups according to baPWV and maxIMT values: patients with “low baPWV and low maxIMT” (n = 279), those with “high baPWV and low maxIMT” (n = 181), those with “low baPWV and high maxIMT” (n = 241), and those with “high baPWV and high maxIMT” (n = 339). As shown in Figure 1C, the cumulative incidence rate of CVE was significantly higher in the patients with “high baPWV and low maxIMT” compared to those with “low baPWV and low maxIMT” (p = 0.030, log-rank test). Although the patients with “low baPWV and high maxIMT” also showed a tendency to have a higher risk as compared to those with “low baPWV and low maxIMT,” it did not reach statistical significance (p = 0.071, log-rank test). Interestingly, the cumulative incidence rate of CVE was significantly higher in the patients with “high baPWV and high maxIMT” compared to the other 3 groups. Furthermore, a multivariate Cox proportional hazards regression model, in which conventional risk factors and baseline therapies were entered, also revealed that both baPWV (HR = 1.33 [95%CI: 1.09-1.62]; p = 0.004) and maxIMT (HR = 1.21 [95%CI: 1.01-1.44]; p = 0.036) were independent predictors for CVE (Table 2).

Similarly, subjects with both “high PWV” and “high IMT” had a significantly (p < 0.05) higher risk of developing the major CVE as compared to those with either “high PWV” or “high IMT,” as well as those with neither (Additional file 4: Figure S1C.) A multivariate Cox proportional hazards regression model, in which FRS, baPWV, and maxIMT were entered, revealed that baPWV was a predictor for the major CVE (HR = 1.61 [95%CI: 1.31-1.97]; p < 0.001) independently of FRS and maxIMT, but maxIMT was marginally associated with the major CVE (HR = 1.19 [95%CI: 1.00-1.42]; p = 0.054) independently of FRS and baPWV (Additional file 1: Table S2).

### Contribution of baPWV and maxIMT in the prediction of cardiovascular events

**Figure 2:**
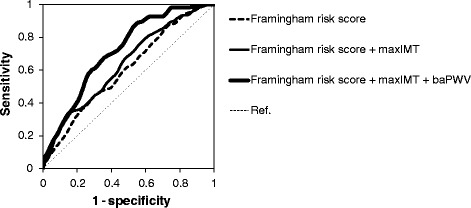
**Time-dependent ROC curves for predicting cardiovascular events**. ROC curves were based on models of the predictability for cardiovascular events with the use of FRS alone; FRS and maxIMT; or FRS, maxIMT, and baPWV. The AUCs for cardiovascular events were 0.60 [95%CI: 0.54-0.67] (FRS alone), 0.63 [95% CI: 0.60-0.82] (FRS and maxIMT), and 0.72 [95%CI: 0.67-0.78] (FRS, maxIMT, and baPWV). The addition of maxIMT alone to FRS resulted in a significant increase in AUC (ΔAUC 0.03 [95% CI: 0.01 to 0.11]; *p* = 0.01). Addition of baPWV to the FRS and maxIMT resulted in a further significant increase in AUC (0.08 [95% CI: 0.01 to 0.11]; p = 0.02).

To examine whether the addition of baPWV and/or maxIMT to conventional risk factors could improve the predictability for CVE, time-dependent ROC curves were plotted (Figure 2). The addition of maxIMT alone to FRS resulted in a significant increase in area under the curve (AUC) (from 0.60 [95%CI: 0.54-0.67] to 0.63 [95%CI: 0.60-0.82]; *p* = 0.01). Notably, the addition of baPWV to the FRS and maxIMT resulted in a further significant increase in AUC (from 0.63 [95%CI: 0.60-0.82] to 0.72 [95%CI: 0.67-0.78]; p = 0.02).

Next, to examine whether the addition of baPWV and/or maxIMT to conventional risk factors could improve the predictability for major CVE, time-dependent ROC curves were plotted. The addition of maxIMT alone to FRS significantly increased AUC (from 0.59 [95%CI: 0.51-0.66] to 0.63 [95%CI: 0.55-0.72]; p = 0.01) and the addition of baPWV to FRS and maxIMT further increased AUC (from 0.63 [95%CI: 0.55-0.72] to 0.72 [95%CI: 0.65-0.78]; p = 0.02) (Additional file 5: Figure S2).

These results suggested that evaluation of baPWV, in addition to maxIMT and conventional risk factors, can substantially improve the ability to identify individuals with a high risk for CVE among asymptomatic type 2 diabetic patients.

## Discussion

Early detection of asymptomatic severe coronary artery disease as well as cerebrovascular disease, and subsequent rapid intervention, are important to reduce mortality in the management of diabetes. Although cardiovascular risk assessment based on the conventional risk factors is recommended for predicting cardiovascular risk, validation studies have shown that this approach had only moderate performance [1-3]. Therefore, a non-invasive and inexpensive tool to predict the risk of subclinical or silent atherosclerosis with more than moderate predictive ability is required for identifying individuals at high-risk of CVE.

Because baPWV is easy to measure and requires no special techniques, it could be suitable for screening in routine care. It has been already shown that baPWV is closely associated with conventional cardiovascular risk factors in subjects with and without diabetes [34]. The present study also demonstrated that there were significant associations between conventional cardiovascular risk factors and baPWV, suggesting that high baPWV itself reflects inherent cardiovascular risk factors. However, there was insufficient evidence of its clinical significance; although several longitudinal studies have demonstrated that higher baPWV levels were associated with increased risk of CVD or mortality among the general population [11,12,17,18] and high-risk patients with coronary heart disease or renal diseases [35-37], it has been unclear whether baPWV level is associated with increased risk of the development of CVE in asymptomatic type 2 diabetic patients.

The present study clearly demonstrated that a higher baPWV level was associated with increased risk of development of CVE in asymptomatic type 2 diabetic patients. Furthermore, a multivariate analysis revealed that baPWV was still an independent predictor for CVE even after adjusting for conventional cardiovascular risk factors. These results were consistent with the findings observed in general populations, and with the idea that diabetic patients with arterial stiffness are prone to develop CVE.

The present study also confirmed the results of several previous studies reporting that carotid IMT, a marker of early atherosclerosis and vascular remodeling, was an independent predictor of CVE in asymptomatic diabetic patients [38-40].

Interestingly, type 2 diabetic subjects with both “high PWV” and “high IMT” had a significantly greater risk of developing CVE compared to those with either “high PWV” or “high IMT”, and to those with neither. Furthermore, a multivariate regression model revealed that both baPWV and maxIMT were significant independent predictors for CVE, even after adjustment for each other and the conventional risk factors. These results suggest that baPWV and maxIMT reflect different aspects of cardiovascular risk that leads to onset of CVE, and that measurement of either or both of these two parameters could substantially improve the risk assessment for future CVE compared with the risk assessment based on conventional cardiovascular risk factors alone.

To test the above hypothesis, the predictability of baPWV and/or maxIMT for identifying patients at high risk for CVE was then evaluated over time. Importantly, measurement of baPWV improved the risk assessment for future CVE when compared with the Framingham Risk Score, which is one of the established risk scores reflecting conventional cardiovascular risk factors. To the best of our knowledge, this is the first study to demonstrate that adding baPWV to an established risk score significantly improved the risk assessment for future CVE in type 2 diabetic patients. The question of whether measurement of carotid IMT can add prognostic information beyond conventional cardiovascular risk markers remains controversial [40-44]. However, we confirmed previous findings that the addition of carotid IMT to conventional risk factors could improve the assessment of risk for future CVE, but the improvement of the predictability was small. Interestingly, the AUC of the time-dependent ROC curve was further increased after the addition of baPWV to FRS and maxIMT (from 0.63 to 0.72; p = 0.02). This is the first study to show that evaluation of the baPWV, in addition to carotid IMT and conventional risk factors, further improved the ability to identify individuals with a high risk for CVE. These findings highlight the clinical usefulness of baPWV in the risk assessment of CVE among asymptomatic type 2 diabetic patients, suggesting that it can be applied more broadly in routine care in this group.

Several limitations of our study should be discussed. First, the sample size and the number of events were relatively small. Furthermore, there was a gender imbalance among participants. Second, the primary outcome was new onset of all cardiovascular events, including the need for coronary revascularization and PAD (soft events). However, even if we focus on new onset of the major CVE, similar results were obtained. Third, the subjects of this study were Japanese patients with type 2 diabetes mellitus and were a cohort with relatively low cardiovascular risk. Although the incidence of CVE observed was comparable to the value reported in a previous Japanese study [45], it was notably lower compared to the incidence observed in Western diabetic populations [46,47]. Therefore, it would be premature to generalize our findings to other racial or ethnic groups or non-diabetic subjects. It should be also noted that the definitions of hypertension and dyslipidemia adopted in this study were for Japanese diabetic patients and somewhat different from those used in Western countries (especially in the thresholds of systolic blood pressure and serum LDL cholesterol level). It may reduce comparability of our study with others concerning the same topic, although we confirmed that the results of the analyses were almost the same even if we adopted Western definitions. Fourth, the development of PAD was considered one of the components of primary outcome. However, some asymptomatic PAD cases might have been overlooked, since ankle-brachial indices were not measured annually during the observation period in some cases. Fifth, in some analyses we divided subjects into two groups according to their baPWV values and compared the between-group differences. However, the cut-off value of baPWV used in these analyses was not based on previous literature but used the median value of this cohort. Sixth, the ROC curve analysis showed that the AUC of FRS for predicting risk of CVE in type 2 diabetic patients would be small. However, this was consistent with those of several previous studies, which also showed a value of around 0.60 [48].

The present study focused on the question of whether measurement of baPWV can provide any useful information for identifying patients with a high risk for CVE at a certain point in their life with diabetes. Our thesis would have been strengthened if it were shown that measurement of baPWV could improve the risk prediction of CVE even after adjustment for the therapeutic regimen during the follow-up period. However, the therapeutic regimens (e.g. types of agents administered and duration of administration) used by our study subjects varied greatly and we were not able to include such a wide range of parameters in the multivariate regression model, because the number of observed CVE was insufficient. Therefore, whether the therapeutic regimens used and/or longitudinal monitoring of major cardio-metabolic parameters during follow-up affects the predictability of baPWV should be evaluated in further studies.

Notwithstanding these limitations, our study indicates that measurement of baPWV can provide useful information for identifying patients with a high risk of CVE.

## Conclusions

The present study suggests that baPWV, a non-invasive and user-friendly method for quantitatively estimating arterial stiffness, can improve the risk prediction of cardiovascular events in asymptomatic type 2 diabetic patients.

## Additional files

Additional file 1: Table S1. Relative risk of cardiovascular events. **Table S2.** Relative risk of major cardiovascular events. Additional file 2: Figure S1A. Kaplan–Meier curves depicting the cumulative probability of major cardiovascular events in patients with baPWV values lower than the median (thin line) (<1550 cm/s, n = 520) and higher than the median (bold line) (≥1550 cm/s, n = 520). The risk for major cardiovascular events was significantly greater in patients with higher baPWV values compared to those with lower baPWV values (*p* = 0.001, log-rank test). Additional file 3: Figure S1B. Kaplan–Meier curves depicting the cumulative probability of major cardiovascular events in patients with maxIMT values lower than the median (thin line) (<1.0 mm, n = 460) and higher than the median (bold line) (≥1.0 mm, n = 580). The risk for major cardiovascular events was significantly greater in patients with higher maxIMT values compared to those with lower maxIMT values (*p* = 0.003, log-rank test). Additional file 4: Figure S1C. Kaplan–Meier curves depicting the cumulative probability of major cardiovascular events in patients with “low baPWV and low maxIMT (baPWV <1550 cm/s and maxIMT <1.0 mm, n = 279)” (thin gray line), “high baPWV and low maxIMT (baPWV ≥1550 cm/s and maxIMT <1.0 mm, n = 181)” (dotted line), “low baPWV and high maxIMT (baPWV <1550 cm/s and maxIMT ≥1.0 mm, n = 241)” (thin black line), and “high baPWV and high maxIMT (baPWV ≥1550 cm/s and maxIMT ≥1.0 mm)” (bold black line) (n = 580). The cumulative incidence rate of major cardiovascular events was significantly greater in the patients with “high baPWV and high maxIMT” compared to the other 3 groups (p < 0.05). Although the patients with “high baPWV and low maxIMT” and those with “low baPWV and high maxIMT” showed a tendency towards a higher risk compared to those with “low baPWV and low maxIMT”, there were no significant differences between the groups. Additional file 5: Figure S2. Time-dependent ROC curves for predicting cardiovascular events. ROC curves were based on models of the predictability for major cardiovascular events with the use of FRS alone; FRS and maxIMT; or FRS, maxIMT, and baPWV. The AUCs for major cardiovascular events were 0.59 [95%CI: 0.51-0.66] (FRS alone), 0.63 [95%CI: 0.55-0.72] (FRS and maxIMT), and 0.72 [95%CI: 0.65-0.78] (FRS, maxIMT, and baPWV). The addition of maxIMT to FRS resulted in significant increase in AUC (ΔAUC = 0.04 [95% CI: 0.00 to 0.09]; p = 0.01). Addition of baPWV to the FRS and maxIMT resulted in a further significant increase in AUC (0.09 [95% CI: 0.02 to 0.15]; p = 0.02).
