# Supplementary material for: Clinical utility of brachial-ankle pulse wave velocity in the prediction of cardiovascular events in diabetic patients
Source: Cardiovasc Diabetol. 2014 Sep 5;13:128. doi: 10.1186/s12933-014-0128-5 (PMC4172854; doi:10.1186/s12933-014-0128-5)
Supplement: Additional file 1: Table S1. — Relative risk of cardiovascular events. Table S2. Relative risk of major cardiovascular events. [file 12933_2014_128_MOESM1_ESM.docx]

Supplementary Table 1 Relative risk of cardiovascular events

|  | HR (95%CI) | *p* value |
| --- | --- | --- |
| Gender (male) | 0.84 (0.58-1.23) | 0.367 |
| Age (years) | 1.08 (1.05-1.10) | <0.001 |
| Smoking habit (yes) | 0.79 (0.55-1.15) | 0.226 |
| Body mass index (kg/m^2^) | 1.02 (0.97-1.07) | 0.443 |
| HbA1c (%) | 1.13 (0.97-1.31) | 0.108 |
| Duration of diabetes (years) | 1.05 (1.02-1.09) | 0.001 |
| Systolic BP (mmHg) | 1.02 (1.01-1.03) | 0.004 |
| Diastolic BP (mmHg) | 1.00 (0.98-1.02) | 0.821 |
| Presence of hypertension (yes) | 1.61 (1.00-2.58) | 0.049 |
| Total cholesterol (mg/dl) | 1.00 (0.99-1.00) | 0.546 |
| HDL cholesterol (mg/dl) | 0.99 (0.98-1.01) | 0.240 |
| LDL cholesterol (mg/dl) | 0.99 (0.99-1.01) | 0.606 |
| Triglyceride (mg/dl) | 1.00 (1.00-1.00) | 0.140 |
| Presence of dyslipidemia (yes) | 0.92 (0.62-1.37) | 0.678 |
| Serum Creatinine (mg/dl) | 1.54 (1.17-2.03) | 0.002 |
| Administration of |  |  |
| Anti-diabetic drugs (%) | 1.31 (0.85-2.02) | 0.222 |
| Anti-hypertensive drugs (%) | 1.31 (0.90-1.90) | 0.153 |
| Anti-hyperlipidemic drugs (%) | 0.93 (0.64-1.36) | 0.722 |
| Framingham Risk Score for CVD (per 1 SD) | 1.40 (1.18-1.65) | <0.001 |

Univariate Cox proportional hazards regression analysis was used.

Supplementary Table 2 Relative risk of major cardiovascular events

|  | HR (95%CI) | *p* value |
| --- | --- | --- |
| baPWV (per 1 SD) |  |  |
| Model 1 | 1.67 (1.39-2.01) | <0.001 |
| Model 2 | 1.67 (1.16-1.81) | <0.001 |
| Model 3 | 1.39 (1.08-1.78) | 0.011 |
| Model 2 + maxIMT | 1.61 (1.31-1.97) | <0.001 |
| Model 3 + maxIMT | 1.41 (1.09-1.82) | 0.008 |
| maxIMT (per 1 SD) |  |  |
| Model 1 | 1.32 (1.13-1.55) | 0.001 |
| Model 2 | 1.30 (1.10-1.55) | 0.002 |
| Model 3 | 1.05 (0.86-1.28) | 0.645 |
| Model 2 + baPWV | 1.19 (1.00-1.42) | 0.054 |
| Model 3 + baPWV | 1.07 (0.87-1.33) | 0.517 |

Cox proportional hazards regression analyses unadjusted and adjusted for the following covariates:

Model 1, unadjusted

Model 2, adjusted for the Framingham Risk Score

Model 3, adjusted for the conventional risk factors and baseline therapies (gender, age, smoking habit, BMI, HbA1c, duration of diabetes, systolic blood pressure, HDL cholesterol, LDL cholesterol, triglyceride, serum Cr levels, administration of hypoglycemic drugs, anti-hypertensive drugs, and anti-hyperlipidemic drugs).
