# Supplementary figures and images for: Clinical utility of brachial-ankle pulse wave velocity in the prediction of cardiovascular events in diabetic patients
Source: Cardiovasc Diabetol. 2014 Sep 5;13:128. doi: 10.1186/s12933-014-0128-5 (PMC4172854; doi:10.1186/s12933-014-0128-5)

## Slide 1
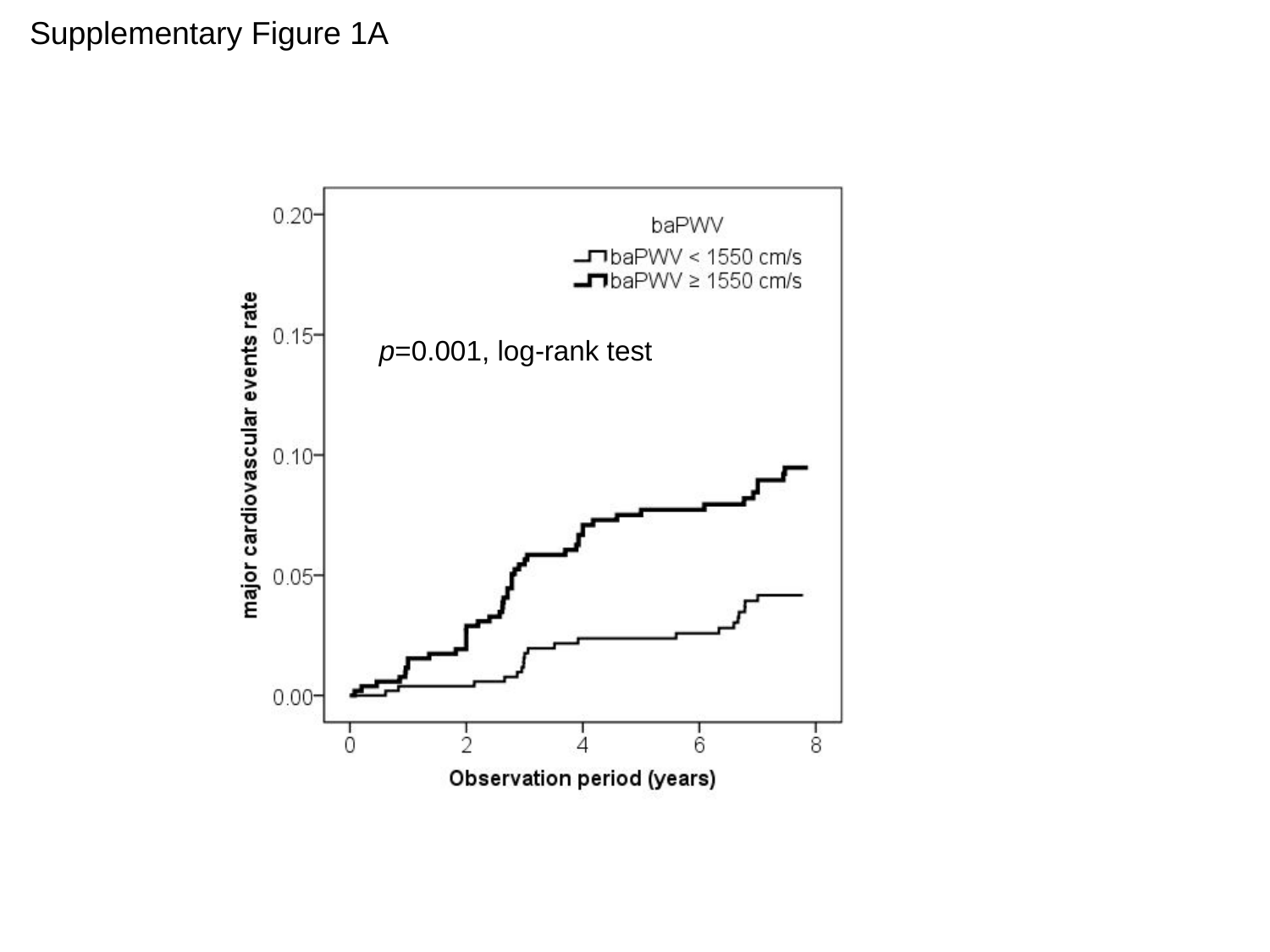

Supplementary Figure 1A
p=0.001, log-rank test

Supplement: Additional file 2: Figure S1A. — Kaplan–Meier curves depicting the cumulative probability of major cardiovascular events in patients with baPWV values lower than the median (thin line) (<1550 cm/s, n = 520) and higher than the median (bold line) (≥1550 cm/s, n = 520). The risk for major cardiovascular events was significantly greater in patients with higher baPWV values compared to those with lower baPWV values (p = 0.001, log-rank test). [file 12933_2014_128_MOESM2_ESM.pptx]

## Slide 1
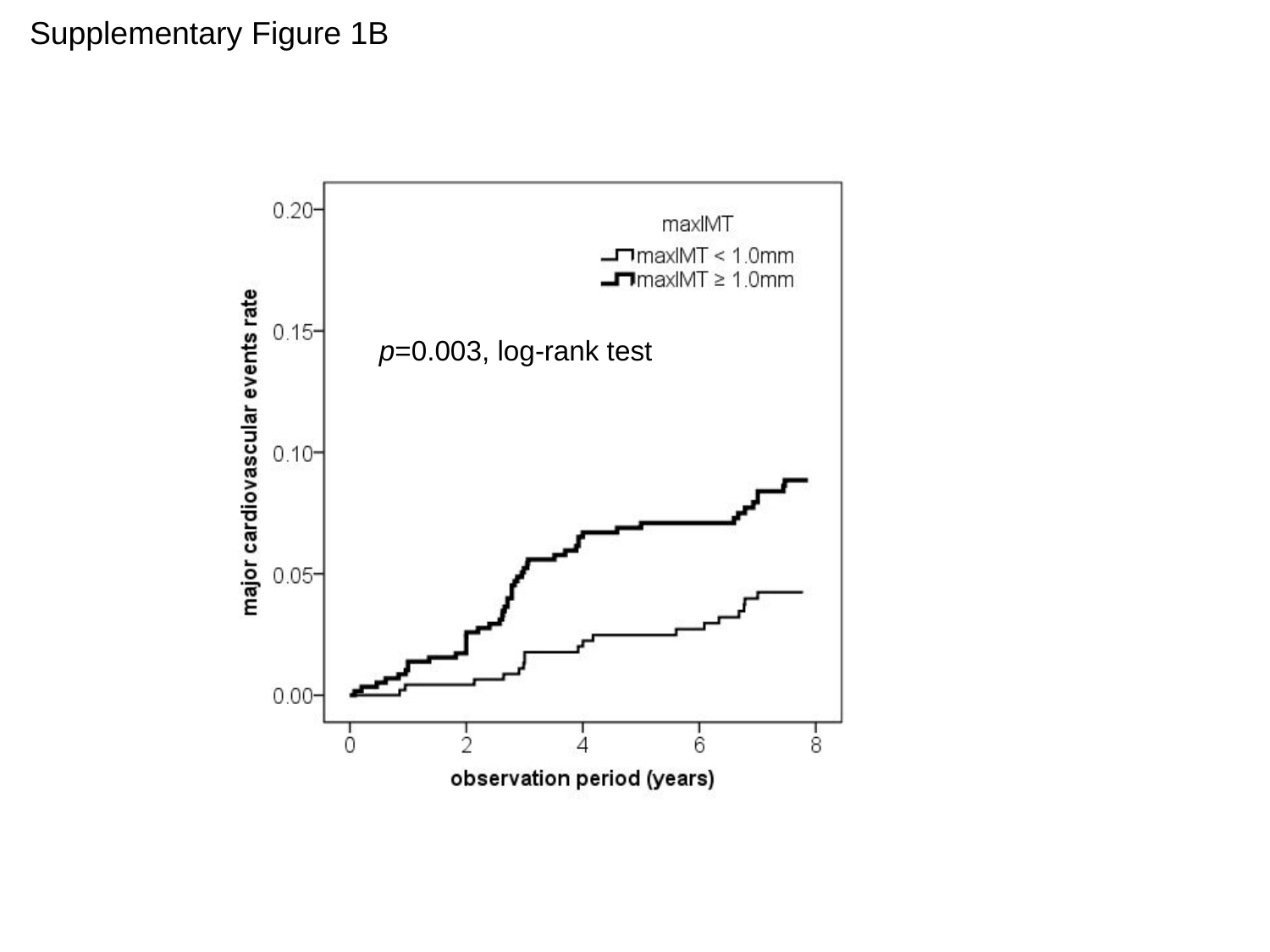

Supplementary Figure 1B
p=0.003, log-rank test

Supplement: Additional file 3: Figure S1B. — Kaplan–Meier curves depicting the cumulative probability of major cardiovascular events in patients with maxIMT values lower than the median (thin line) (<1.0 mm, n = 460) and higher than the median (bold line) (≥1.0 mm, n = 580). The risk for major cardiovascular events was significantly greater in patients with higher maxIMT values compared to those with lower maxIMT values (p = 0.003, log-rank test). [file 12933_2014_128_MOESM3_ESM.pptx]

## Slide 1
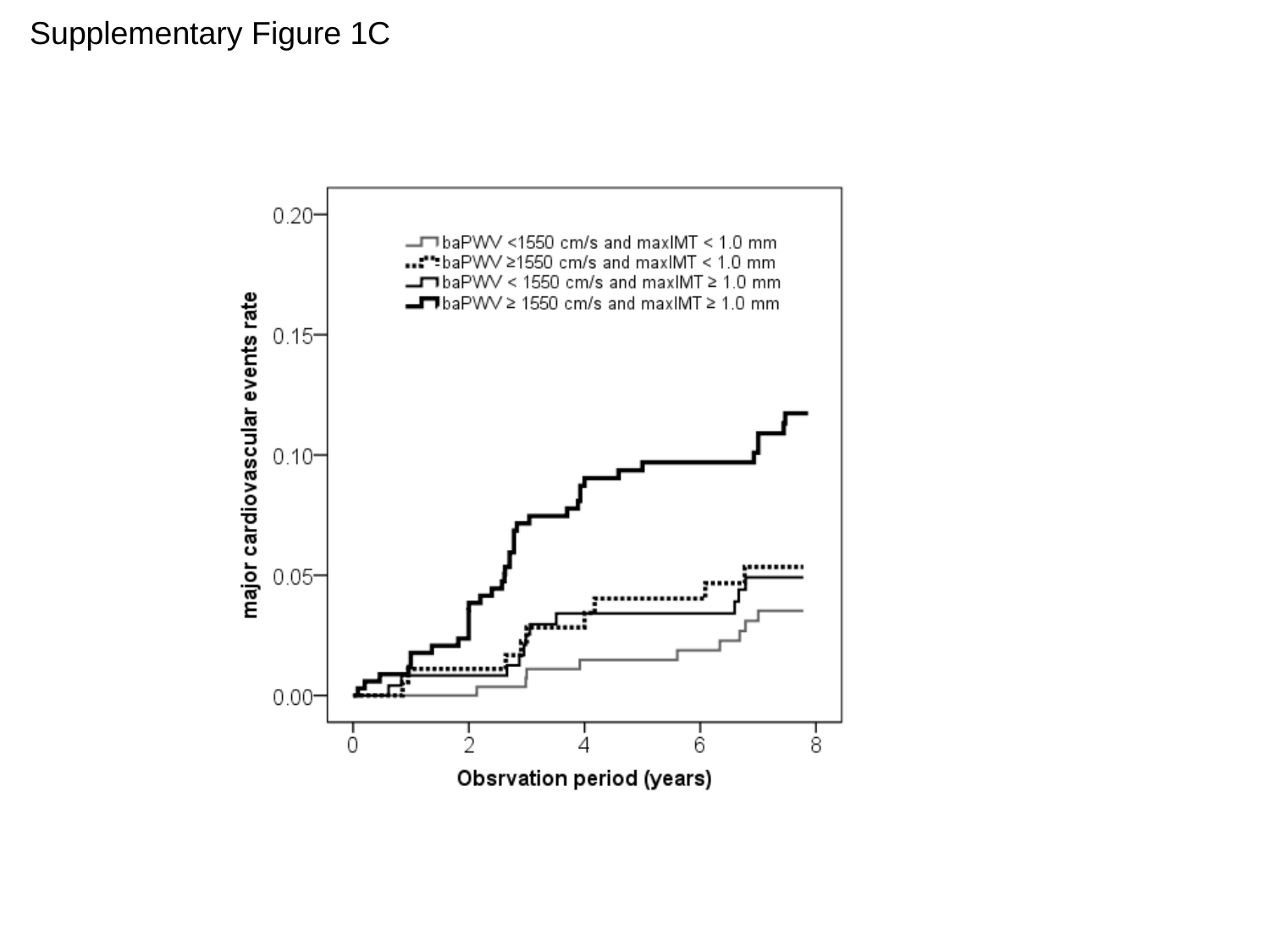

Supplementary Figure 1C

Supplement: Additional file 4: Figure S1C. — Kaplan–Meier curves depicting the cumulative probability of major cardiovascular events in patients with “low baPWV and low maxIMT (baPWV <1550 cm/s and maxIMT <1.0 mm, n = 279)” (thin gray line), “high baPWV and low maxIMT (baPWV ≥1550 cm/s and maxIMT <1.0 mm, n = 181)” (dotted line), “low baPWV and high maxIMT (baPWV <1550 cm/s and maxIMT ≥1.0 mm, n = 241)” (thin black line), and “high baPWV and high maxIMT (baPWV ≥1550 cm/s and maxIMT ≥1.0 mm)” (bold black line) (n = 580). The cumulative incidence rate of major cardiovascular events was significantly greater in the patients with “high baPWV and high maxIMT” compared to the other 3 groups (p < 0.05). Although the patients with “high baPWV and low maxIMT” and those with “low baPWV and high maxIMT” showed a tendency towards a higher risk compared to those with “low baPWV and low maxIMT”, there were no significant differences between the groups. [file 12933_2014_128_MOESM4_ESM.pptx]

## Slide 1
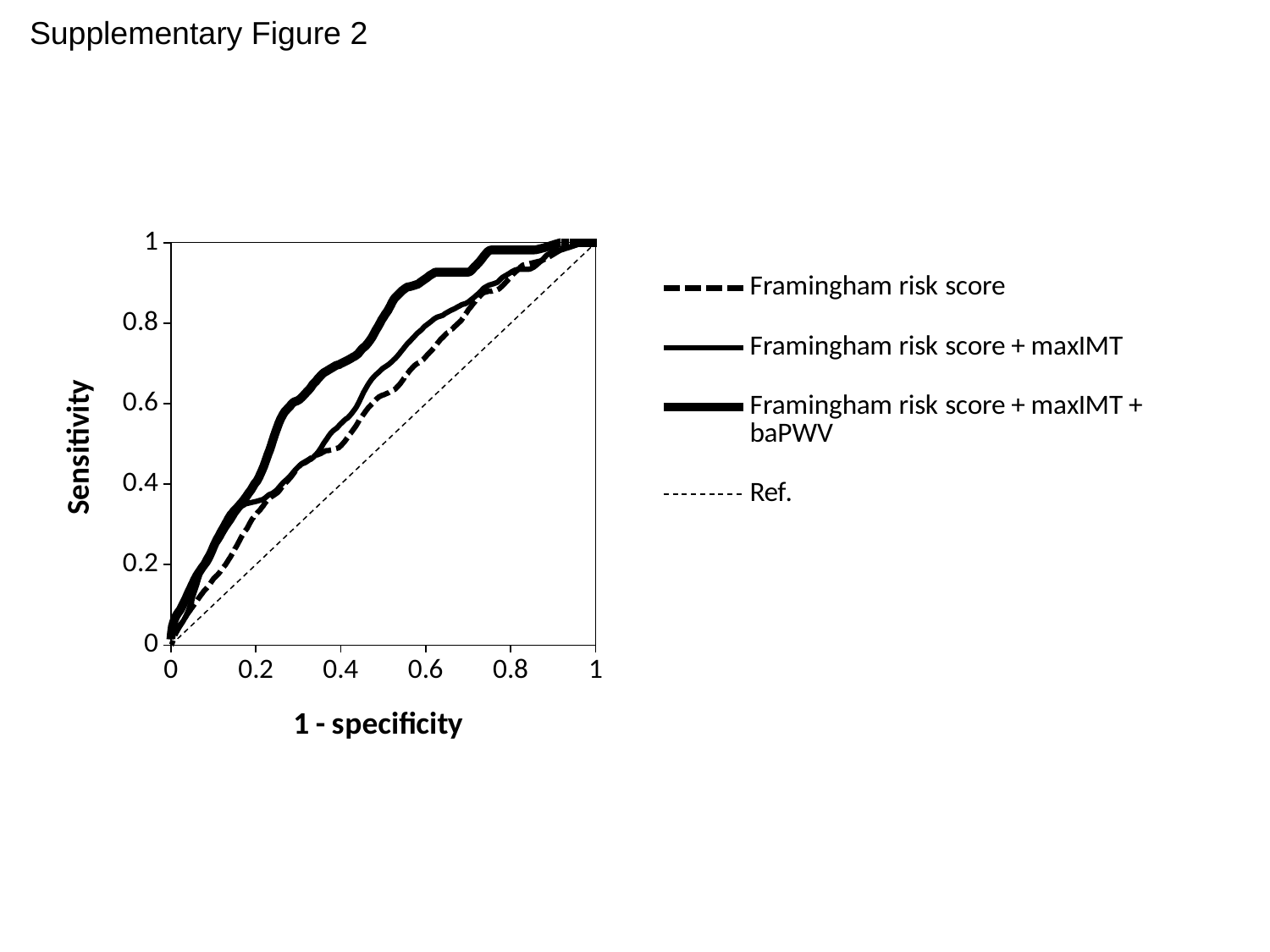

Supplementary Figure 2
### Chart
| Category | | | | |
|---|---|---|---|---|

Supplement: Additional file 5: Figure S2. — Time-dependent ROC curves for predicting cardiovascular events. ROC curves were based on models of the predictability for major cardiovascular events with the use of FRS alone; FRS and maxIMT; or FRS, maxIMT, and baPWV. The AUCs for major cardiovascular events were 0.59 [95%CI: 0.51-0.66] (FRS alone), 0.63 [95%CI: 0.55-0.72] (FRS and maxIMT), and 0.72 [95%CI: 0.65-0.78] (FRS, maxIMT, and baPWV). The addition of maxIMT to FRS resulted in significant increase in AUC (ΔAUC = 0.04 [95% CI: 0.00 to 0.09]; p = 0.01). Addition of baPWV to the FRS and maxIMT resulted in a further significant increase in AUC (0.09 [95% CI: 0.02 to 0.15]; p = 0.02). [file 12933_2014_128_MOESM5_ESM.pptx]
